# Buffer strips can pre-empt extinction debt in boreal streamside habitats

**DOI:** 10.1186/1472-6785-13-24

**Published:** 2013-07-10

**Authors:** Ville A O Selonen, Janne S Kotiaho

**Affiliations:** 1 Department of Biological and Environmental Science, University of Jyväskylä, PO Box 35, 40014, Finland

**Keywords:** Conservation, Extinction debt, Forest management, Legislation, Valuable habitat, Woodland key habitat

## Abstract

**Background:**

Conservation of biological diversity and economical utilization of natural resources form an almost inevitable confrontation between the two. In practice, however, a balance between the two ought to be found, and in managed boreal forests, preservation of woodland key habitats is increasingly used strategy to safeguard biological diversity. According to the Finnish Forests Act, certain Forest Act habitat (FAH) types must be safeguarded, provided they are clearly distinguishable from their surroundings. Furthermore, once the habitat has been identified as a FAH, its special characteristics must not be altered. Both of these aspects contain ambiguities that potentially undermine the practical application of the Act. We designed a replicated sampling study to address these ambiguities at the most common FAH type, riparian habitat of small boreal streams. As response variables we used vascular plants and mosses. We asked i) how wide is the FAH around small streams that is distinguishable from its surrounding and ii) how wide buffer strip around the FAH is sufficient for long term to preserve the natural species community composition of the FAH.

**Results:**

We found that an average three meters wide strip around the stream constitutes the distinguishable FAH and that a minimum of 45 meters wide buffers on both sides of the stream are needed for the species community composition to remain unaltered.

**Conclusions:**

We conclude that 45 meters wide buffers appear sufficient to safeguard vascular plant and moss species communities within the FAH, prevent local populations from extinctions and thus pre-empt extinction debt that would be realised with more narrow buffers. While 45 meters may seem intolerable from the commercial forestry point of view, anything less than that may be intolerable from the point of view of conservation, and thus against the idea of sustainable use of natural resources.

## Background

Conservation of biological diversity and economical utilization of natural resources form an almost inevitable confrontation between the two. In boreal forests, biodiversity and commercial forestry are the key players. Today, the negative effects of forestry on forest biodiversity are axiomatic [1,2], and in many countries practical measures have been initiated to remedy and overcome these effects. In Fennoscandia and Baltic countries, one measure that has been taken is to preserve the so called woodland key habitats (WKH) in the commercial forests [3]. WKHs are small habitat patches with presumably high conservation value [3] and they are generally perceived to be a cost-effective tool in conservation of commercial forest biodiversity. Although the ecology behind the concept is questioned and criticized [2,4,5], in practice WKHs are widely applied in Fennoscandia and Baltic countries. There is some variation among the countries in the details of the definitions and in protection of the WKHs [3], but the underlying idea in all is to preserve habitat patches that are thought to be of value from the standpoint of forest ecosystems and biodiversity.

In Finland, a Forest Act was passed in 1996, the main aim of which is to allow sustainable management and utilization of forests, while simultaneously safeguarding biodiversity [6]. In the Finnish Forest Act, the concept of WKH was applied and some habitat types were defined as Forest Act Habitats (FAH) where demanding, rare and threatened species are likely to occur [6]. All of these habitats are terrestrial and the most numerous FAH type is the riparian habitat of the boreal brooks or rivulets (small streams) [7]. Riparian habitats are a heterogeneous mosaic of terrestrial and aquatic habitats, therefore often harbouring a rich biodiversity [8-10]. Small streams themselves and the adjacent riparian habitats appear to be vulnerable and their biodiversity is often adversely influenced by forests management [11,12].

Two important practical details in the Finnish Forest Act are that first, to qualify as a FAH that must be protected, the habitat must be clearly distinguishable from its surroundings [6], and second, once the habitat has been identified as a FAH, its special characteristics must not be altered. Both of these details contain ambiguity that potentially undermines the practical application of the Act: what constitutes clearly distinguishable or special characteristics and how to ensure that the special characteristics are not altered. Moreover, no clear guidelines for the delineation and demarcation exist. The decision about the distinguishability, the definition of special characteristics and the overall demarcation of the FAH depends on the forest authorities on site, e.g. during the management planning.

The most likely biological aspect that can make a habitat clearly distinguishable from its surroundings for a human observer is variation in the plant community composition. In similar line of thought, the special characteristics that must not be altered are the characteristics of the plant community. It is well established that forest management influences plant community composition [13-15]. Therefore, since FAH’s special characteristics must not be altered, unmanaged buffer strips around the FAHs are of fundamental importance for the spirit of the Act. Research on edge effects has provided robust evidence that communities of the target habitat will be altered if the buffer strips are not sufficient [16,17]. More specifically, studies that have focussed on plant communities of boreal forests emphasize the importance of the width of the buffer strips [18,19], and it has been stated that if the plant communities of the streams and the streamside riparian habitats are to be preserved, sufficient buffer strips are necessary [20-22]. Unfortunately, from the practical point of view it is not enough to state that sufficient buffer strips are needed. Rather, we need to provide clear guidelines based on solid empirical evidence for the widths of buffer strips that constitute sufficient and are likely to be enough to protect the community composition in the long run. However, what should be born in mind is that from society’s perspective, over buffering may be as undesirable as under buffering since in commercial forests the management must be economically as well as ecologically sustainable and the apparent trade-off is rather challenging.

From these grounds we designed a study to address two concrete and practical issues: i) how wide is the Forest Act Habitat around small streams that is distinguishable from its surrounding and ii) how wide buffer strip around the stream is sufficient to preserve the natural species community composition of the FAH.

## Methods

### Study sites and sampling design

We established 39 study sites (out of 213 candidate sites) on riparian FAHs located in mature managed spruce dominated coniferous forests, in Central Finland within a 100 kilometre radius of the city of Jyväskylä (62.23°N, 25.74°E). Our design uses space-for-time-substitution. This method has the advantage that it provides us with the opportunity to analyse patterns based on a single field season. At the same time, the disadvantage of the method is that it relies on the assumption that the sites are similar to begin with, and that for any given management combination the successional trajectories of the sites would be similar. While the latter is not possible to control for in a study design, a violation of this assumption would make any patterns due to management more difficult to observe. As we do observe patterns (see Results) it is likely that this assumption is not badly violated. The former assumption is easier to control in the study design and to this end we applied selection criteria to the study sites to make them as similar as possible: All selected sites belong to the southern boreal vegetation zone, and in all sites the forests were mature, managed and spruce dominated coniferous forests, characterized by deciduous undergrowth. Due to the previous management history large deciduous trees are rare and we included in the study only sites in which they were completely absent. According to the Finnish forest site type classification [23], the vegetation on the streamside was mainly the *Oxalis-myrtillus* type (OMT, herb-rich heath forest) with occasional patches of *Myrtillus* (MT) and *Oxalis Maianthemum* (OMaT) types. In addition, some peatland vegetation type occurred occasionally, thus the parent material in the soil was varying between the peat and till. All sites were selected to be non-flooding and topographically homogenous. In addition, all other habitat factors (e.g. boulders, stand characteristics and deadwood) were taken into account and sites were preselected to be as similar as possible (see Additional file 1: Appendix 1). The edge orientation was observed and north facing edges were not selected (see Additional file 1: Appendix 1 and 2). All water channels were small and narrow (on average of one meter) streams or rivulets with regular, year-round flow. Seven sites were considered as unmanaged reference sites, where the nearest clear-cut was located at least 80 meters from the focal site. Although these seven sites are considered here as unmanaged, their forest management history apart from the location of the nearest clear-cut is similar to all other sites. The unmanaged and managed sites did not differ from each other in any of the measured habitat characteristics except that the diameter and height of the trees at unmanaged reference sites were on average slightly less (differences were <5cm and <4 meters respectively) than those at the managed sites (Additional file 1: Appendix 1 and 2). Differences are a result of that unmanaged sites had higher amount of smaller trees that was not been thinned out yet. Given that the stand ages and in particular total volumes did not differ, we consider these statistically significant differences biologically trivial. It is worth noting that our unmanaged reference sites will be more disturbed than would be pristine sites, had there been any available for the study. Thus our results pointing to impacts of harvesting on FAH species communities should be considered as minimum estimates.

Among the 32 managed study sites, the distance from the stream to the clear-cut (i.e. width of the FAH plus the buffer strip) varied from 0 to 50 meters. The time since the forest behind the buffer strip was harvested by clear-cutting varied from 1 to 50 years. All buffer strips were one-sided and the other side of the stream was equivalent to unmanaged sites (minimum of 80 meters to the closest clear-cut). All of the sites were inventoried during 2003-2004. Each study site consisted of three parallel species sampling lines orthogonally from the stream shoreline, the distance of which were between 10-15 meters from the other. Each sampling line was divided into one square meter sampling units. From the shoreline up to 15 metres each of the sampling units were inventoried. After the first 15 meters, the sampling was conducted every 5 meters to the clear-cut. In each sampling unit, ground layer’s vascular plants (excl. arborescent species) and ground layer’s mosses (Bryophyta) were identified to the species and the coverage determined as percentages. Species crowing distinctly above the ground layer (e.g. on a boulder or dead wood) were excluded from the sampling.

### How wide is the FAH around small streams that is distinguishable from its surrounding

The immediate stream side is by Forest Act definition part of the FAH. Therefore, to empirically determine the extent or width of the riparian FAH that is distinguishable for the surrounding forests, we rearranged our species community data of the seven unmanaged sites by the distance from the stream and ran an analysis of similarities (ANOSIM) between the distances [24]. ANOSIM is a non-metric analysis based on dissimilarity measures and it uses the rank order of dissimilarity values, thus being analogous to non-metric multidimensional scaling (NMDS). We performed ANOSIM with vascular plant and moss species data separately and with a pooled vascular plant and moss species data. This ensures comprehensive interpretation of the delineation. In ANOSIM Bray-Curtis similarity index was used. Bray-Curtis similarity index takes into account the relative abundance of species and, in addition to changes in species identities, reveals also changes in species community composition that are due to changes in the relative abundances of species. Prior to analysis, species data was log_10_-transformed to downweight the dominant taxa. Species that occurred only once were excluded from the analysis. Significances of similarities between groups were derived from 10000 permutations. We compared the community composition of our focal sampling unit bordering the stream (sampling unit one) to each of the community compositions of the other sampling units 2-15 meters from the stream. ANOSIM was performed with PAST (version 2.08) [25].

### How wide buffer strip around the stream is sufficient to preserve the natural species community composition of the FAH

The value obtained from the above analysis on the unmanaged sites was used to determine the extent of the FAH on the managed sites. Sites with a buffer strip less than the extent of the FAH (3 meters, see Results) were excluded from the forthcoming analyses. Thus, the final number of managed sites used in the analyses is 20.

Regression analysis was conducted to determine differences in species richness (i.e. number of species) and taxonomic diversities [26] between different management histories (i.e. width of the buffer strip and time since harvested). Suitability of variables for analysis was verified and required transformations conducted. Taxonomic diversity was determined to obtain a variable to reflect the changes in species composition. Taxonomic diversity is an index describing distribution of abundances and taxonomic relatedness of species in each of the studied sites. It is a combination of standard diversity indices and an average relatedness between any two species chosen at random from the site [26]. Taxonomic diversity in one sample is $\Delta =\frac{\sum \sum_{i<J}\omega_{\mathrm{ij}x_{i}x_{j}}}{\sum \sum_{i<j}x_{i}x_{j}+\sum_{i}\frac{x_{i}\left(x_{i}-1\right)}{2}}$ , where the ω_ij_ is weight (ω_ij_ = 0 if *i* and *j* are the same species, ω_ij_ = 1 if they are the same genus, ω_ij_ = 2 if they are the same family, etc. according to the desired taxonomic categories). The *x* denotes the abundances of species *i* and *j*. In other words, the measure weights species depending on their affinity (i.e. near kinship species are weighted less). The higher the taxonomic diversity is, the more different taxonomic categories sample encompasses and more diverse the species assemblage is. The suggested advantage of this measure is that it attempts to capture phylogenetic diversity and is more closely linked to functional diversity than the more traditional diversity indices [26,27]. It is suggested that such phylogenetic diversity indices should be used as a biodiversity metric for predicting and monitoring of biodiversity changes and threats [28].

To intensify the taxonomic information, vascular plant and moss species data was specified with family data according to Hämet-Ahti et al.1998 [29] and Ulvinen et al. 2002 [30], respectively. In this analysis, the abundance of a certain species is the number of occupied sampling units in a site. The buffer strip width and time since harvested were log-transformed (log_10_(1+x)). Species richness and the taxonomic diversity were determined with PAST (version 2.08) [25] and all statistical analyses were carried out with PASW 18 (SPSS Inc.).

Analysis of similarities (ANOSIM) was used to determine the minimum buffer width for no change in community composition in the FAH. First we divided managed sites into six groups (every fifth meter 1-5, 6-10 etc.), and then compared the species community composition of each of the managed groups to unmanaged sites. The buffer width from where the species community composition in the managed sites no longer differed from that of the unmanaged sites indicates the minimum buffer strip width for maintaining the community composition.

It can be considered that the environment in which a species is most abundant is close to the species environmental optimum [31,32]. The estimate of the optimum environment can be calculated as a weighted average of the environmental variable values of the sites in which the species is present [32]. The weighted average estimate of the optimum is ${\tilde{u}}_{k}=\frac{\sum_{i=1}^{n}x_{\mathrm{ik}}y_{i}}{\sum_{i=1}^{n}x_{\mathrm{ik}}}$, where *x* is the abundance of species *k* in sample *i* and *y* is the empirical environmental value in sample i (e.g. buffer width or time since harvested). In the present study, this method was used to estimate the optimum buffer width and the optimum time since harvested for species in the FAH. Estimate was calculated for species observed in five or more sites. In addition, we used the method in the unmanaged sites to estimate the “natural” optimum distance of the species from the stream. Optimum values were determined with PAST (version 2.08) [25].

## Results

### How wide is the FAH around small streams that is distinguishable from its surrounding

In vascular plants, the community compositions of the sampling units 5-15 were significantly different from our focal sampling unit (Table 1). This means that the habitat strip of about 0-4 meters from the stream is distinguishable from the surrounding forest. In mosses the corresponding distinguishable habitat strip was 0-2 meters and in the combined data of vascular plants and mosses it was 0-3 meters from the stream (Table 1). Naturally, each species has its own characteristic ecological requirements. Therefore, based on species specific abundance, we have tabulated the natural optimal distance of vascular plant and moss species from the stream in Additional file 1: Appendix 3 and 4 respectively.

**Table 1:** Analysis of community similarities (ANOSIM) between the first sampling unit (1 meter from the stream) and the other sampling units 2–15 meters from the stream

	**Vascular plants**		**Mosses**		**Pooled data**	
**Comparison**	**R**	**p**	**R**	**p**	**R**	**p**
1 - 2	-0.069	0.778	0.050	0.275	-0.066	0.751
1 - 3	0.064	0.244	0.272	**0.012**	0.086	0.166
1 - 4	0.122	0.072	0.474	**0.000**	0.271	**0.008**
1 - 5	0.317	**0.002**	0.621	**0.001**	0.460	**0.001**
1 - 6	0.402	**0.001**	0.632	**0.000**	0.518	**0.000**
1 - 7	0.469	**0.002**	0.608	**0.000**	0.502	**0.001**
1 - 8	0.507	**0.001**	0.658	**0.001**	0.528	**0.001**
1 - 9	0.563	**0.001**	0.689	**0.001**	0.559	**0.001**
1 - 10	0.599	**0.001**	0.703	**0.001**	0.583	**0.001**
1 - 11	0.610	**0.001**	0.693	**0.001**	0.600	**0.001**
1 - 12	0.655	**0.001**	0.739	**0.000**	0.631	**0.001**
1 - 13	0.645	**0.001**	0.772	**0.001**	0.640	**0.001**
1 - 14	0.656	**0.001**	0.708	**0.001**	0.635	**0.000**
1 - 15	0.661	**0.000**	0.770	**0.001**	0.651	**0.000**

Data is from the unmanaged reference sites. R values are effect sizes based on the difference of mean ranks between and within groups [32]. N for all groups is 7.

### How wide buffer strip around the stream is sufficient to preserve the natural species community composition of the FAH

In the previous analysis, the distinguishable habitat strip was determined to extend 3 meters from the stream. From now on, we only analyse diversity in this distinguishable habitat strip and call it the FAH, the special characteristics of which must not be altered by forest management. After excluding sites with the buffer strip less than the width of the FAH, the final number of managed sites included into the analysis was 20. Total number of vascular plant and moss species found in the whole study area were 130 and 85, respectively. The total number of vascular plant and moss species found in the FAH were 108 and 69, respectively and the number of unique vascular plant and moss species for FAH were 19 and 21 respectively. The total number of vascular plant and moss species found outside the FAH were 92 and 56, respectively and the number of unique vascular plant and moss species for outside the FAH were 19 and 9 respectively.

**Figure 1:**
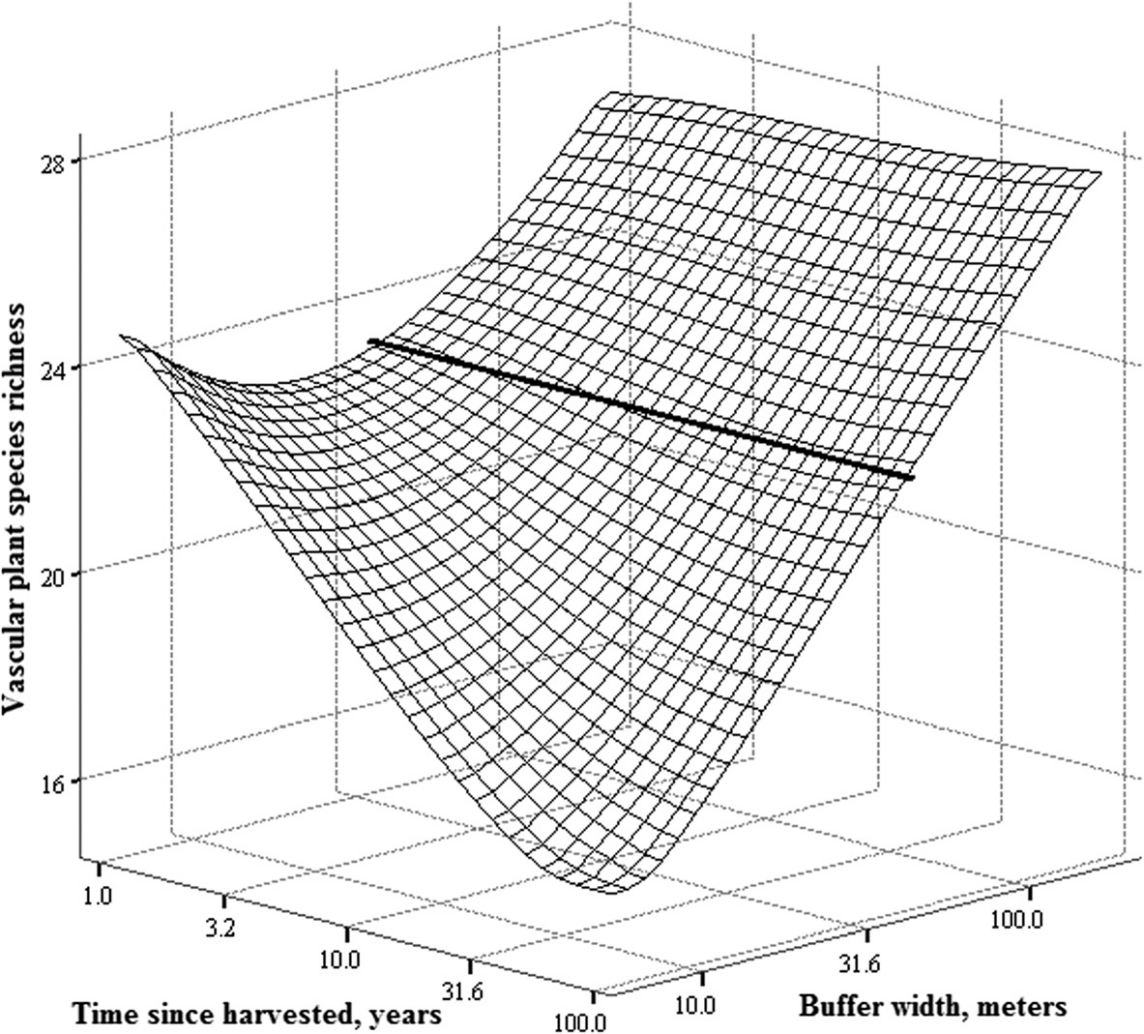
**Interaction between the width of the buffer strip and time since harvested on vascular plant species richness.** Bold line across the surface represents the 45-meters wide buffer. Vascular plant species data is from the FAH. Buffer strip width and time since harvested are at log_10_-scale.

**Figure 2:**
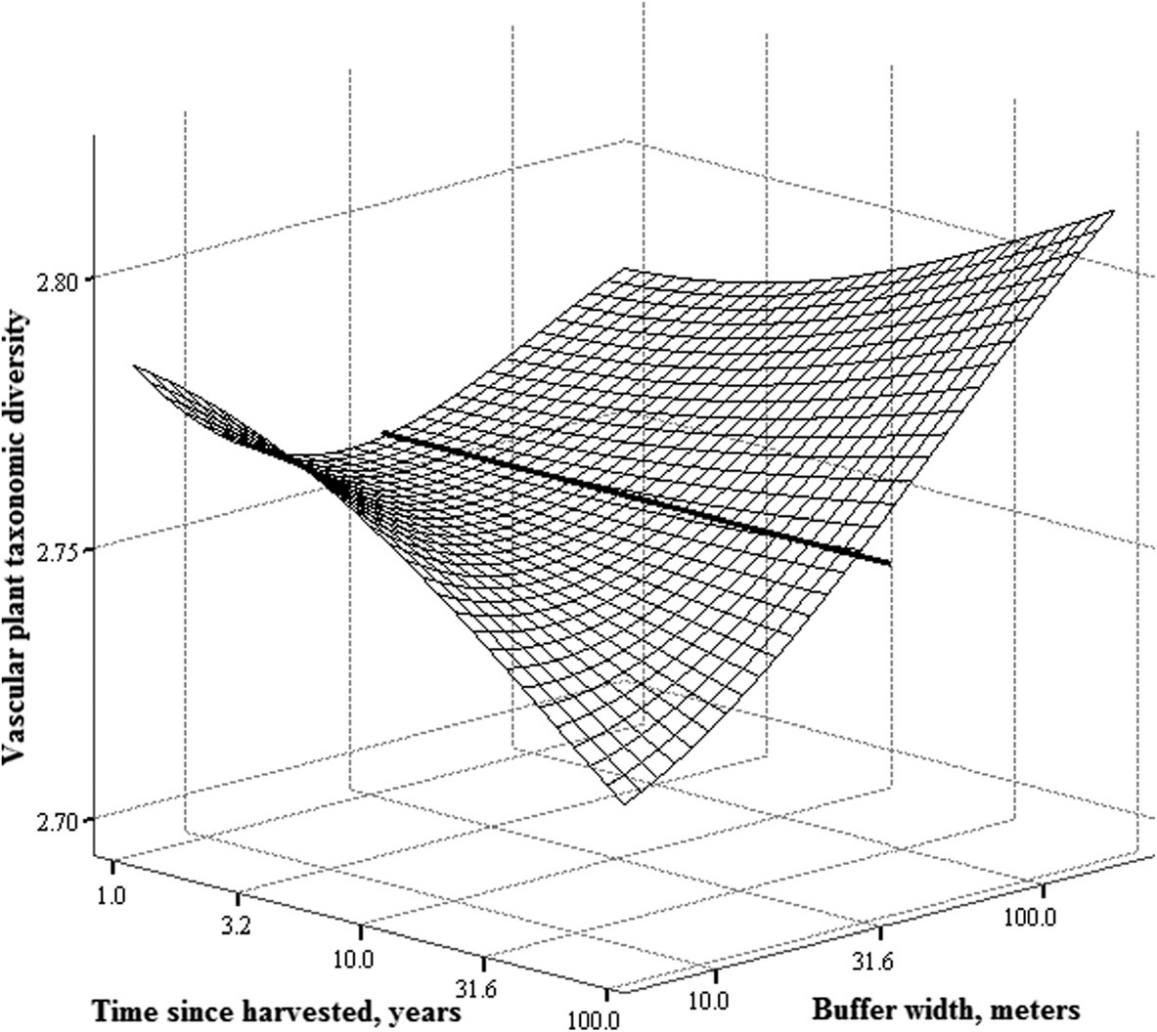
**Interaction between the width of the buffer strip and time since harvested on taxonomic diversity of vascular plants.** Bold line across the surface represents the 45-meters wide buffer. Vascular plant species data is from the FAH. Buffer strip width and time since harvested are at log_10_-scale.

There was an interaction between the width of the buffer strip and the time since harvested by clear-cutting on both, the vascular plant species richness and taxonomic diversity of FAH (Table 2). We have depicted the interactions in Figures 1 and 2, from which one can see that on a narrow buffer strips both species richness and taxonomic diversity of vascular plants decline with time, while on wider buffer strips similar decline does not occur.

**Table 2:** Regression analyses for vascular plant and moss species richness and taxonomic diversity

		**R**^**2**^	**F**	**Sig.**	**Partial η**^**2**^
Buffer*Time	Plant species	0.489	5.822	**0.024**	0.202
	Plant diversity	0.270	4.427	**0.047**	0.161
	Moss species	0.220	0.918	0.348	0.038
	Moss diversity	0.301	2.889	0.103	0.112
Buffer	Moss species	0.189	0.598	0.447	0.024
	Moss diversity	0.213	6.220	**0.020**	0.206
Time	Moss species	0.189	5.568	**0.027**	0.188
	Moss diversity	0.213	0.051	0.823	0.002

Buffer*Time is the interaction term, i.e. the product term of variables. Buffer and Time denotes variables buffer width and the time since harvested, respectively. Plant and moss species denotes species richness (number of species) and diversities are taxonomic diversities. Values are calculated from log_10_-transformed data. Species data is from the FAH. Degrees of freedom for the models are 1, 24.

To estimate the minimum buffer width needed to safeguard the FAH vascular plant community composition, we divided the buffer widths into six classes and compared the vascular plant species community of each with the corresponding communities of unmanaged reference sites with ANOSIM. FAH community composition differed from the unmanaged references still with 36 meters wide buffers and the communities were unaltered only after the buffer strip widths exceeded 45 meters (Table 3). It is noteworthy that although the sample size and thus power to observe a significant difference for the last comparison was small, the effect size R also decreases ten-fold indicating a real change towards more similar communities. Vascular plant species benefiting from anthropogenic disturbance were found in FAHs with narrow buffer strips (see Additional file 1: Appendix 5). In these species, the smallest weighted average of buffer widths was 0 meters, i.e. not at all FAH external buffer (Additional file 1: Appendix 5).

**Table 3:** **Analysis of community similarity (ANOSIM) of different buffer width categories between unmanaged (N**_**1**_**) and managed sites (N**_**2**_**)**

		**Vascular plants**		**Mosses**	
**Buffer width (metres)**	**N**_**1**_**, N**_**2**_	**R**	**p**	**R**	**p**
0 - 5	7, 3	0.758	**0.009**	0.677	**0.008**
6 - 10	7, 3	0.651	**0.010**	0.544	**0.008**
11 - 15	7, 5	0.488	**0.001**	0.390	**0.008**
21 - 25	7, 4	0.442	**0.019**	0.454	**0.011**
32 - 36	7, 3	0.540	**0.025**	0.482	**0.009**
45 - 50	7, 2	0.058	0.410	0.451	0.084

Species communities are from the FAH.

**Figure 3:**
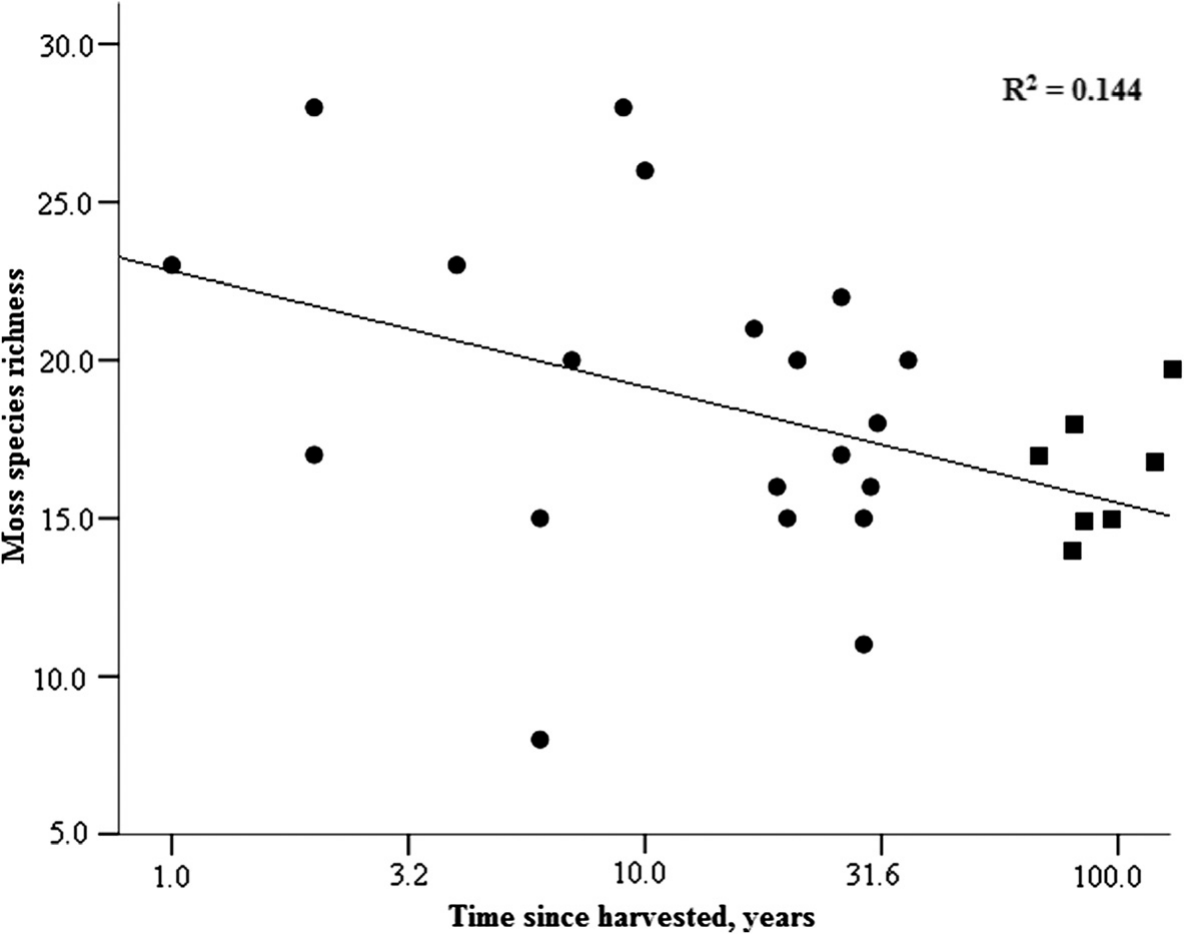
**The effect of time since harvested on moss species richness.** Moss species data is from the FAH. Circles and squares denote managed and unmanaged sites, respectively. Time axis is at log_10_-scale.

**Figure 4:**
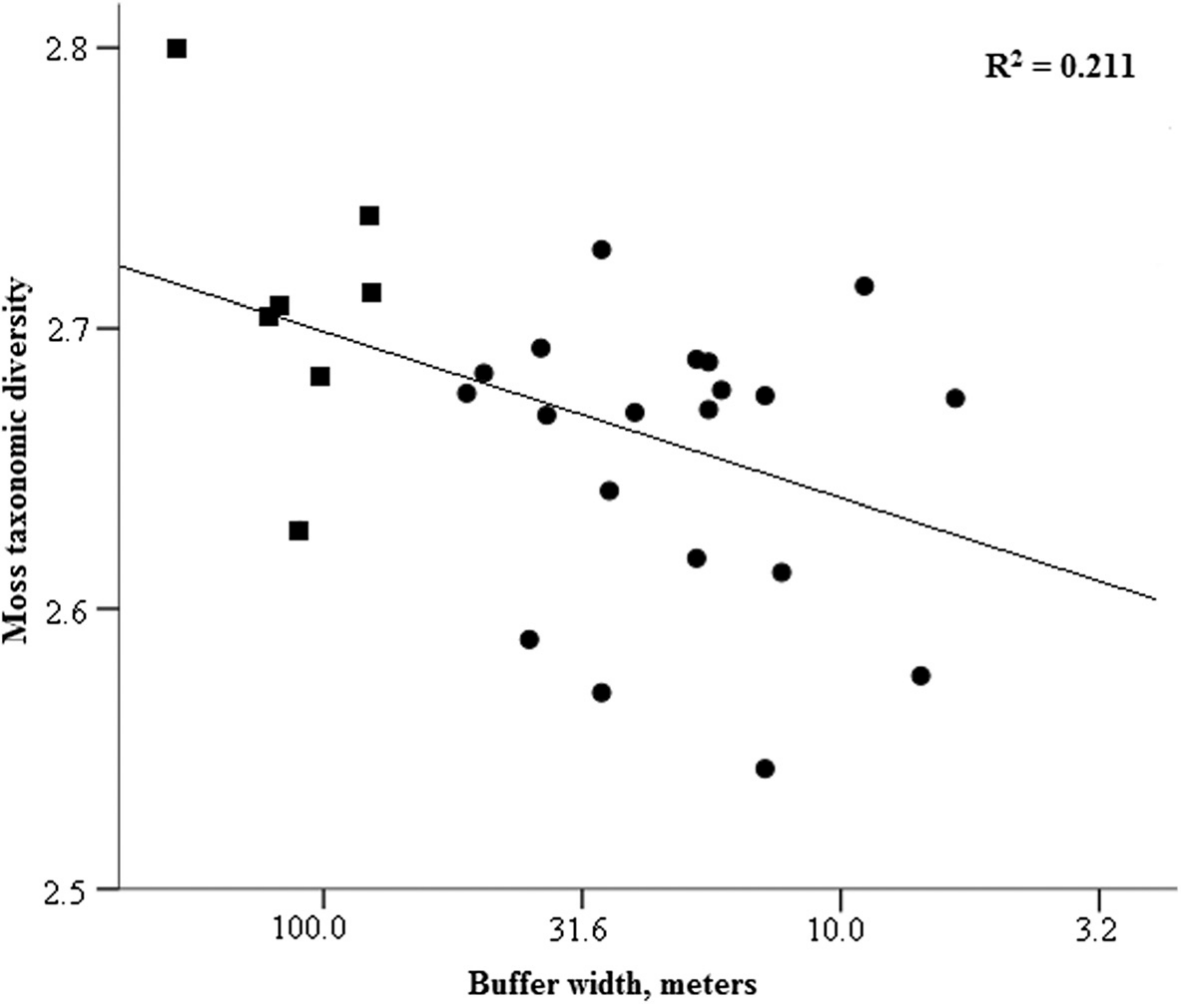
**The effect of width of buffer strip on the taxonomic diversity of mosses.** Moss species data is from the FAH. Circles and squares denote managed and unmanaged sites, respectively. Buffer width axis is reverse and at log_10_-scale.

For moss species richness or taxonomic diversity there were no interaction between the width of the buffer strip and the time since harvested (Table 2). However, moss species richness declined with time since harvested, whereas the taxonomic diversity of mosses increased with the buffer width (see Table 2 and Figures 3 and 4). Similar to vascular plants, also moss community compositions of the FAH differed from the unmanaged references still with 36 meters wide buffers and the moss communities appeared unaltered only after the buffer strip widths exceeded 45 meters (Table 3). However, in mosses the effect size does not change much for the last comparison suggesting that the change in the significance is related to the decreased sample size and even with 45 meters wide buffers the communities may still have been altered. As with vascular plants, moss species benefiting from anthropogenic disturbance were found in the FAH with narrow buffer (see Additional file 1: Appendix 6). However, in mosses, the smallest weighted average of buffer widths was 12 meters (Additional file 1: Appendix 6).

## Discussion

Our first objective was to determine how wide is the Forest Act Habitat (FAH) around small streams that is distinguishable from its surrounding. Although defining an average width of streamside riparian habitat or FAH may be ecologically questionable and undesirable, from the management point of view such a generalisation is essential. Despite the importance of the generalization for management, it may not be stressed too much that care is needed when such a generalization is executed, and in practise every delineation has to be done individually depending on forests stand structure, vegetation type and topography of the site. Thus, even if our result that the FAH was on average 3 meters wide strip along the small stream is correct, the actual metric value should be decided on site.

Buffer strips are not mentioned in the Forest Act, but what is important, is that the Forest Act states that the characteristics of the FAH may not be altered. Therefore, to fulfil the statutes of the Act, it is necessary that a buffer strip around the 3 meters wide FAH must be left. From these premises, our second objective was to determine how wide buffer strip around the stream is needed to preserve the natural species community composition of the FAH. We found that vascular plant species richness and taxonomic diversity were affected by an interaction between buffer strip width and time since the formation of the buffer strip. At narrow buffer strips, species richness and diversity declined with time but similar decline did not occur in the wider buffer strips. In mosses there were no interactions, but the moss species richness declined with time since harvested by clear-cutting and the taxonomic diversity declined with the declining width of the buffer strip.

The interaction between buffer strip width and time since harvested on vascular plant species richness and taxonomic diversity provides an indication of extinction debt [33-35] due to forest management. Although the effect sizes were at most medium (Table 2), the implication for the local risk of extinction due to forest management are not trivial. In our study, the FAH itself was not directly disturbed by the management, but still, extinction debt was accrued in the FAH depending on the distance (i.e. buffer strip width) from the disturbance. Based on figure 1, it appears that with narrow buffer strips it takes approximately 10 years to lose 20% of vascular plant species and 30 years for a third of the species to be lost while similar decline in species richness is not evident with wider buffers. With taxonomic diversity (Figure 2) it appears that the decline in FAHs with narrow buffers may be slower and a clear decline is observed only 20-30 years after the disturbance. Decline in taxonomic diversity due to forest management is distressing because taxonomic diversity inflect the ecosystem functioning [28,36], and may be as significant threat to ecosystem services as the much worried climate change [37].

When we are considering the effects of anthropogenic disturbance, it must be remembered that ultimately from the nature conservation perspective, any change on the species community, a loss or a gain of a species, is undesirable. Thus, since the legislation states that characteristics of the valuable habitats may not be altered, any alteration due to forest management that can be detected in the community composition should be considered to violate the act. Based on our analyses, the sufficient width for a buffer strip that pre-empts the creation of extinction debt seems to be around 45 metres. Our sample size for this particular comparison is small, but it is worth noting that from the point of view of conservation, the value of 45 meters can be considered a conservative minimum estimate. This is because all comparisons with less than 45 meter buffer width resulted in a significant difference between the communities.

There are obvious economical costs associated with leaving buffers and thus from the economical point of view one needs to be careful not to over buffer. Above we stated that the 45 meters was a conservative minimum estimate from the point of view of conservation. Thus from the perspective of sustainable forest management it should be clear that this is indeed the minimum that must be left in order to avoid altering the community compositions, while it is not clear that buffering more than 45 meters would not benefit the biodiversity even more. Ours is not the first study to suggest that at least 45 meters wide buffers should be left but earlier values of similar magnitude have been suggested [38-41].

The interesting and somewhat natural result of mosses, the increasing diversity with increasing buffer width, is result of functional buffering. The wider buffers are able to safeguard the species diversity of the FAH. Moreover, the result of decreasing species richness with time is indicating that due to management actions there are a loss of species that will occur with a time lag. Species that have short optimum time since harvesting and wide optimum buffer strip width are species that are still present just after the management, but soon disappear in spite of relatively wide buffers.

We did not observe a clear increase in the vascular plant species richness after the disturbance, although based on earlier research such an increase could have been expected [14,15]. However, some indication of such an effect may be seen in the figure 1 and 2 if we concentrate on the corner of the matrix where the buffer strip width is small and time since harvested short: in both graphs this corner tends towards higher richness. Moreover, at the FAH we observed a few pioneer plant species typically found in clear-cuts (e.g. *Epilobium angustifolium)* (see Additional file 1: Appendixes 5 and 6). For example, *E. angustifolium* was not present in any of the unmanaged reference sites but was present on the managed sites. On managed sites, weighted averages of the buffer width and time since harvested for occurrences of *E. angustifolium* in the FAH was 0 meters and 5 years, respectively.

The smallest average optimum buffer width for moss species was 9 meters. This species is a pioneer species *Ceratodon purpureus* and it is typically found in clear-cuts. The other common pioneer species *Pohlia nutans* is still found from FAH with up to on average 14 meters wide buffers (Additional file 1: Appendix 6). Overall, occurrence of these kinds of pioneer species in the FAH is evidence for the adjacent disturbance breaking through the narrow buffer strips. Moreover, although species richness of mosses did not rise after clear-cutting, it was the initially species rich stream side habitat that was the main interest, not the typical forests habitat. This leads to already higher richness at the outset compared to the former inland forest orientated studies. Note that the gradual change from stream side species to clear-cut species does not necessarily increase species richness, and ultimately the changes in species community composition in time reveals the effects of the disturbance.

In general the species specific responses to buffer width and time since harvested suggest that some microclimatic changes have taken place in the FAH. In vascular plants, *Moneses uniflora*, typical in moist forests, is found in FAHs with on average 34 meters wide buffers, but only on average one year after the harvesting (see Additional file 1: Appendix 5). Moreover, the moss *Sphagnum riparium*, a species which is known to suffer from drainage, is found on average just one year after harvesting, indicating that it may really suffer from forest management. The average buffer width for *S. riparium* is as much as 34 meters. Similarly another moss species, *Ptilium crista-castrensis*, which is typically found in moist and shady habitat, is found only few years after harvesting (see Additional file 1: Appendix 6). These examples suggest considerable change in moisture conditions and alteration in run-off properties in streamside FAH even with relatively wide buffers. Similarly also exposure to sun and wind is likely to increase evaporation, thus changing ground level moisture and climate conditions. Thus it is clear that the effects of forest management can travel far and buffers of at least 45 meters are needed if we really want to adhere to the statement (and spirit) of the Forest Act that the characteristics of the FAH may not be altered.

## Conclusions

Trying to find a one-size-fits-all –buffer strip width as we did here, is not sensible in ecological sense, simply because organisms with different ecology will respond differently. However, at the same time in reality we very much need to be able to make every day decisions in our commercial forests about how wide buffers to leave. Therefore, we need to work out practicable guidelines for forests managers and authorities that unavoidably overlook some of the ecological detail in the landscape.

Based on our analyses the width of the FAH along the boreal forest streams (sensu Finnish forest Act) is on average 3 meters wide. In practice, it will be safer to use 4-meter FAHs based on vascular plant species. However, as the forest act also demands that the characteristics of these habitats may not be altered, it should immediately be obvious that a buffer is needed. Our results indicate that even if the valuable habitat itself is only a narrow strip along the stream, to conserve this strip unaltered it is imperative to leave a minimum of 45 meters wide buffer strip of forest on both sides of the streams. If we really mean what we have written into the legislation, anything less than 45 meters buffers around the valuable habitats will be against the spirit of the act, and against the idea of sustainable use of natural resources.

## Abbreviations

ANOSIM: Analysis of similarities; FAH: Forest Act habitat; WKH: Woodland key habitat.

## Competing interests

Neither of the authors has competing interests.

## Authors’ contributions

Authors conceived the study and the design together. VS conducted and coordinated the field work and the species identification. Together the authors conducted the statistical analysis and wrote the manuscript. Both authors read and approved the final manuscript.

## Supplementary Material

Additional file 1 **Appendix 1.** Stand characteristics of studied sites. **Appendix 2.** Test statistics between managed and unmanaged reference sites: i) T-test for equality of habitat and stand characteristic means; ii) Mardia-Watson-Wheeler test for equal directional distribution (i.e. direction of edge in managed sites and direction of sample lines in unmanaged reference sites); iii) Pearson correlation between independent variables used in regression analysis. **Appendix 3.** Optimum distance (meters) of vascular plant species from the stream. Optimum distances are based on weighted averaging. Data is from the unmanaged reference sites. **Appendix 4.** Optimum distance (meters) of moss species from the stream. Optimum distances are based on weighted averaging. Data is from the unmanaged reference sites. **Appendix 5.** Optimum buffer width (meters) and time from harvesting (years) for vascular plant species. Optimums are based on weighted averaging. Species are from the FAH. **Appendix 6.** Optimum buffer width (meters) and time from harvesting (years) for moss species. Optimums are based on weighted averaging. Species are from the FAH. Click here for file
