# Supplementary material for: Buffer strips can pre-empt extinction debt in boreal streamside habitats
Source: BMC Ecol. 2013 Jul 10;13:24. doi: 10.1186/1472-6785-13-24 (PMC3716660; doi:10.1186/1472-6785-13-24)
Supplement: Additional file 1 — Appendix 1. Stand characteristics of studied sites. Appendix 2. Test statistics between managed and unmanaged reference sites: i) T-test for equality of habitat and stand characteristic means; ii) Mardia-Watson-Wheeler test for equal directional distribution (i.e. direction of edge in managed sites and direction of sample lines in unmanaged reference sites); iii) Pearson correlation between independent variables used in regression analysis. Appendix 3. Optimum distance (meters) of vascular plant species from the stream. Optimum distances are based on weighted averaging. Data is from the unmanaged reference sites. Appendix 4. Optimum distance (meters) of moss species from the stream. Optimum distances are based on weighted averaging. Data is from the unmanaged reference sites. Appendix 5. Optimum buffer width (meters) and time from harvesting (years) for vascular plant species. Optimums are based on weighted averaging. Species are from the FAH. Appendix 6. Optimum buffer width (meters) and time from harvesting (years) for moss species. Optimums are based on weighted averaging. Species are from the FAH. [file 1472-6785-13-24-S1.pdf]

Appendix 1. Stand characteristics of studied sites.

Site	Buffer width	Time since harvested	Direction of edge	Slope m/10m	Peat, cm	Average stand age, years	Average diameter, cm	Average Stand height, m	Stand volume m3/ha
Site 1	39	28	130	1	20	80	26	23	294
Site 2	6	5	160	0.5	0	90	27	23	192
Site 3	48	20	250	1	0	80	27	22	154
Site 4	18	25	210	1	10	100	30	25	255
Site 5	18	35	230	1	30	86	27	24	223
Site 6	0	25	80	1	10				
Site 7	0	26	230	0	20				
Site 8	19	2	120	2	20	100	35	30	158
Site 9	17	8	250	2	5	81	26	24	267
Site 10	13	28	80	1.5	10	78	24	18	182
Site 11	2	12	250	2	0	100	35	30	158
Site 12	16	1	220	1	0	80	24	20	200
Site 13	1	5	210	0.5	0				
Site 14	12	18	80	1	0	80	22	19	224
Site 15	1	3	190	0.5	10				
Site 16	50	3	200	0.5	10	85	27	22	244
Site 17	8	16	310	0	0	80	24	18	183
Site 18	2	24	70	0.5	0	90	27	23	192
Site 19	0	30	80	1.5	15				
Site 20	5	29	110	1.5	0	81	23	22	191
Site 21	37	1	190	1.5	0	85	27	22	244
Site 22	28	19	80	1	0	100	26	21	223
Site 23	3	4	210	0	10	113	31	24	331
Site 24	36	0	80	0.5	15	100	30	23	253
Site 25	27	6	110	0.5	0	100	26	21	223
Site 26	17	9	250	0.5	10	99	27	23	338
Site 27	28	25	210	0.5	0	80	29	24	345
Site 28	1	4	250	1	10				
Site 29	41	21	110	0.5	30	85	26	21	216
Site 30	24	30	220	0.5	0	113	30	23	331
Site 31	0	18	260	1.5	10				
Site 32	13	5	310	0.5	30	100	26	20	243
Ref. 1	125	84	190	1.5	0	84	20	17	200
Ref. 2	169	79	210	0.5	10	79	23	19	301
Ref. 3	315	105	250	0.5	20	105	22	18	231
Ref. 4	210	67	120	1.5	0	67	25	19	258
Ref. 5	125	109	220	1	0	139	21	18	197
Ref. 6	200	96	170	2	0	96	21	18	233
Ref. 7	185	80	120	0.5	10	80	24	21	351

Appendix 2. Test statistics between managed and unmanaged reference sites: i) T-test for equality of habitat and stand characteristic means; ii) Mardia-Watson-Wheeler test for equal directional distribution (i.e. direction of edge in managed sites and direction of sample lines in unmanaged reference sites); iii) Pearson correlation between independent variables used in regression analysis.

i)	t-test				
	MD	SE	df	t	p
Slope m/10m	-0.181	0.238	37	-0.758	0.453
Peatlayer thickness, m	0.029	0.039	37	0.733	0.468
Average stand age, yr	-2.217	6.156	30	-0.36	0.721
Average diameter cm (1.3m)	4.962	1.276	30	3.888	0.001
Average stand height, m	3.989	1.132	30	3.522	0.001
Tree stand volume	-19.112	24.556	30	-0.778	0.442

ii)	Mardia-Watson-Wheeler test			
	Site mean	Reference Mean	W	p
Direction	185.36	184.05	2.3842	0.30359

iii)	Pearson Correlation		
	N	R	p
Time vs. width	32	-0.071	0.698

Appendix 3. Optimum distance (meters) of vascular plant species from the stream. Optimum distances are based on weighted averaging. Data is from the unmanaged reference sites.

Plant species	Optimum
Pyrola minor	1
Pyrola rotundifolia	1
Carex acuta	2
Scutellaria galericulata	2
Peucedanum palustre	2
Matteuccia struthiopteris	2
Potentilla palustris	2
Lysimachia vulgaris	2
Lysimachia thyrsiflora	3
Epilobium palustre	3
Moneses uniflora	3
Phalaris arundinacea	3
Caltha palustris	3
Callitriche palustris	4
Valeriana sambucifolia	4
Filipendula ulmaria	4
Rubus arcticus	5
Cardamine amara	5
Galium palustre	5
Carex canescens	6
Viola palustris	6
Phegopteris connectilis	6
Agrostis canina	6
Calamagrostis purpurea	6
Athyrium filix-femina	6
Angelica sylvestris	6
Deschampsia cespitosa	7
Carex disperma	7
Geum rivale	7
Ranunculus repens	7
Paris quadrifolia	7
Equisetum sylvaticum	7
Melampyrum pratense	7
Veronica chamaedrys	8
Oxalis acetosella	8
Crepis paludosa	8
Orthilia secunda	8
Carex globularis	8
Trientalis europaea	8
Dryopteris carthusiana	8
Convallaria majalis	8
Luzula pilosa	8

Maianthemum bifolium	8
Gymnocarpium dryopteris	8
Stellaria longifolia	8
Vaccinium vitis-idaea	8
Rubus saxatilis	8
Lycopodium annotinum	9
Melampyrum sylvaticum	9
Vaccinium myrtillus	9
Deschampsia flexuosa	9
Linnaea borealis	9
Juncus filiformis	10
Calamagrostis arundinacea	10
Solidago virgaurea	10
Melica nutans	10
Epilobium montanum	10
Carex digitata	10
Veronica officinalis	11
Goodyera repens	12
Fragaria vesca	12
Viola riviniana	12
Potentilla erecta	13
Lathyrus vernus	14
Geranium sylvaticum	15

Appendix 4. Optimum distance (meters) of moss species from the stream. Optimum distances are based on weighted averaging. Data is from the unmanaged reference sites.

Moss species	Optimum
Calliergonella cuspidata	1
Campylium stellatum	1
Drepanocladus aduncus	1
Fissidens osmundoides	1
Pogonatum urnigerum	1
Sphagnum papillosum	1
Plagiothecium denticulatum	1
Brachythecium rivulare	2
Plagiothecium succulentum	2
Atrichum undulatum	3
Polytrichastrum formosum	3
Plagiomnium cuspidatum	4
Calliergon cordifolium	4
Ptilium crista-castrensis	4
Tetraphis pellucida	4
Rhizomnium punctatum	5
Plagiomnium ellipticum	5
Sphagnum fallax	5
Sphagnum riparium	5
Straminergon stramineum	6
Pohlia nutans	6
Sanionia uncinata	6
Rhizomnium magnifolium	6
Sphagnum squarrosum	6
Pseudobryum cinclidioides	6
Climacium dendroides	6
Sphagnum girgensohnii	6
Plagiothecium laetum	7
Mnium hornum	7
Rhizomnium pseudopunctatum	7
Rhytidiadelphus triquetrus	7
Dicranum fuscescens	7
Sphagnum angustifolium	8
Rhodobryum roseum	8
Polytrichum juniperinum	8
Polytrichum commune	8
Cirriphyllum piliferum	8
Dicranum scoparium	8
Dicranum polysetum	9
Pleurozium schreberi	9
Hylocomium splendens	9
Dicranum majus	9

Aulacomnium palustre	10
Rhytidiadelphus subpinnatus	10
Plagiomnium medium	11
Sphagnum capillifolium	11

Appendix 5. Optimum buffer width (meters) and time from harvesting (years) for vascular plant species. Optimums are based on weighted averaging. Species are from the FAH.

Plant species	Buffer	Time
Epilobium angustifolium	0	5
Potentilla erecta	1	29
Lysimachia thyrsiflora	1	5
Stellaria longifolia	3	5
Lychnis flos-cuculi	3	5
Potentilla palustris	4	15
Equisetum arvense	5	16
Rubus arcticus	7	10
Calamagrostis canescens	7	7
Lysimachia vulgaris	7	8
Peucedanum palustre	8	18
Ranunculus repens	8	12
Paris quadrifolia	9	18
Calamagrostis purpurea	9	16
Fragaria vesca	9	18
Melica nutans	9	16
Epilobium palustre	9	5
Equisetum hyemale	9	18
Carex nigra	10	22
Filipendula ulmaria	11	11
Viola palustris	13	14
Rubus chamaemorus	13	14
Geranium sylvaticum	13	1
Galium palustre	14	13
Equisetum pratense	14	8
Deschampsia cespitosa	15	13
Viola riviniana	15	35
Agrostis canina	15	16
Carex globularis	15	19
Pyrola minor	15	1
Gymnocarpium dryopteris	16	16
Deschampsia flexuosa	16	17
Solidago virgaurea	16	23
Carex digitata	16	26
Lycopodium annotinum	16	12
Caltha palustris	16	11
Dryopteris carthusiana	17	16
Scutellaria galericulata	17	9
Linnaea borealis	17	18
Rubus idaeus	17	17

Angelica sylvestris	18	19
Trientalis europaea	18	13
Phegopteris connectilis	18	14
Carex canescens	18	15
Vaccinium vitis-idaea	18	16
Melampyrum sylvaticum	19	15
Maianthemum bifolium	20	16
Athyrium filix-femina	20	12
Matteuccia struthiopteris	21	30
Equisetum sylvaticum	21	14
Oxalis acetosella	21	15
Geum rivale	21	22
Calamagrostis arundinacea	22	27
Carex disperma	22	5
Rubus saxatilis	23	17
Vaccinium myrtillus	23	16
Luzula pilosa	26	17
Carex loliacea	29	24
Orthilia secunda	29	9
Moneses uniflora	34	1
Juncus effusus	36	28
Convallaria majalis	45	20

Appendix 6. Optimum buffer width (meters) and time from harvesting (years) for moss species. Optimums are based on weighted averaging. Species are from the FAH.

Moss species	Buffer	Time
Ceratodon purpureus	9	18
Plagiothecium curvifolium	10	28
Plagiothecium cavifolium	12	9
Brachythecium rivulare	12	10
Warnstorfia exannulata	14	8
Rhytidiadelphus subpinnatus	14	8
Paraleucobryum longifolium	14	9
Sphagnum capillifolium	14	9
Climacium dendroides	14	9
Pohlia nutans	14	12
Calliergon cordifolium	14	12
Aulacomnium palustre	15	12
Polytrichastrum formosum	15	15
Sphagnum fallax	15	16
Polytrichum commune	15	19
Polytrichastrum longisetum	15	25
Pseudobryum cinclidioides	16	15
Sphagnum girgensohnii	16	17
Rhizomnium pseudopunctatum	17	12
Plagiomnium medium	17	16
Sanionia uncinata	18	9
Dicranum fuscescens	18	12
Straminergon stramineum	18	21
Dicranum scoparium	19	10
Plagiothecium denticulatum	19	16
Rhizomnium punctatum	19	16
Sphagnum squarrosum	20	13
Brachythecium sp.	20	15
Polytrichum juniperinum	20	15
Plagiothecium laetum	20	16
Sphagnum angustifolium	20	18
Atrichum undulatum	20	22
Plagiomnium cuspidatum	21	11
Dicranum polysetum	21	12
Hylocomium splendens	21	14
Dicranum majus	21	16
Pleurozium schreberi	21	16
Rhodobryum roseum	22	23
Rhytidiadelphus triquetrus	23	13
Sphagnum papillosum	23	18

Plagiomnium ellipticum	30	11
Campylium stellatum	30	13
Rhizomnium magnifolium	32	13
Ptilium crista-castrensis	34	1
Sphagnum riparium	34	1
Tetraphis pellucida	34	1
